# Electrical Stimulation of Low-Threshold Proprioceptive Fibers in the Adult Rat Increases Density of Glutamatergic and Cholinergic Terminals on Ankle Extensor α-Motoneurons

**DOI:** 10.1371/journal.pone.0161614

**Published:** 2016-08-23

**Authors:** Olga Gajewska-Woźniak, Kamil Grycz, Julita Czarkowska-Bauch, Małgorzata Skup

**Affiliations:** Department of Neurophysiology, Nencki Institute of Experimental Biology, 3 Pasteur Street, Warsaw, Poland; University of Sydney, AUSTRALIA

## Abstract

The effects of stimulation of low-threshold proprioceptive afferents in the tibial nerve on two types of excitatory inputs to α-motoneurons were tested. The first input is formed by glutamatergic Ia sensory afferents contacting monosynaptically α-motoneurons. The second one is the cholinergic input originating from V0_c_—interneurons, located in lamina X of the spinal cord, modulating activity of α-motoneurons via C-terminals. Our aim was to clarify whether enhancement of signaling to ankle extensor α-motoneurons, via direct electrical stimulation addressed predominantly to low-threshold proprioceptive fibers in the tibial nerve of awake rats, will affect Ia glutamatergic and cholinergic innervation of α-motoneurons of lateral gastrocnemius (LG). LG motoneurons were identified with True Blue tracer injected intramuscularly. Tibial nerve was stimulated for 7 days with continuous bursts of three pulses applied in four 20 min sessions daily. The Hoffmann reflex and motor responses recorded from the soleus muscle, LG synergist, allowed controlling stimulation. Ia terminals and C-terminals abutting on LG-labeled α-motoneurons were detected by immunofluorescence (IF) using input-specific anti- VGLUT1 and anti-VAChT antibodies, respectively. Quantitative analysis of confocal images revealed that the number of VGLUT1 IF and VAChT IF terminals contacting the soma of LG α-motoneurons increased after stimulation by 35% and by 26%, respectively, comparing to the sham-stimulated side. The aggregate volume of VGLUT1 IF and VAChT IF terminals increased by 35% and by 30%, respectively. Labeling intensity of boutons was also increased, suggesting an increase of signaling to LG α-motoneurons after stimulation. To conclude, one week of continuous burst stimulation of proprioceptive input to LG α-motoneurons is effective in enrichment of their direct glutamatergic but also indirect cholinergic inputs. The effectiveness of such and longer stimulation in models of injury is a prerequisite to propose it as a therapeutic method to improve inputs to selected group of α-motoneurons after damage.

## Introduction

There is an accumulation of the experimental data indicating therapeutic potential of modulation of proprioceptive input to α-motoneurons (MNs) by means of electrical stimulation of low-threshold muscle afferents in peripheral nerves. The Hoffmann (H) reflex, an analog of monosynaptic stretch reflex, is a useful tool to control the strength of stimulation addressed predominantly to Ia afferents. When combined with operant conditioning this method allows teaching animals to increase an amplitude of H-reflex of ankle extensor MNs, as shown by Wolpaw’s group (reviewed by Chen and co-authors [1]). It was also shown to compensate the asymmetrical deficit of locomotion, which developed after unilateral lesion of the lateral column in the spinal cord [2]. Importantly, in trained animals, an increase of H-reflex was found to be persistent and the effect was observed up to 100 days after termination of the operant conditioning [3]. These observations point to profound plasticity of the spinal cord induced by proprioceptive stimulation. However, studies aiming to disclose synaptic changes in α-MNs of triceps surae (TS) resulting from the operant conditioning, which lead to an increase of the H-reflexes in the monkey TS muscles, did not bring conclusive results [4] and mechanisms of these changes have not been fully elucidated.

Reorganization of spinal cord circuit induced by low-threshold proprioceptive stimulation requires neurotrophin 3 (NT3). NT3 is involved in maintenance of glutamatergic Ia terminals on the ankle extensor α-MNs [5, 6] and is known to enhance excitatory transmission [7–9]. Chronic stimulation addressed predominantly to low-threshold muscle afferents (Ia) in the tibial nerve of the rat caused clear increase of expression of NT3 in the spinal cord and soleus muscle, as shown by us recently [10]. Therefore, it is reasonable to expect, that increased expression of NT3 after chronic stimulation of these afferents is a good predictor of synaptic plasticity in spinal circuit of extensor MNs, which might result in enrichment of glutamatergic innervation and increased signaling to these MNs. In view of these data, in the current study we aimed to examine how the same paradigm of stimulation, which caused an increase of NT3, will affect glutamatergic low-threshold proprioceptive input to α-MNs of the lateral gastrocnemius (LG).

In addition to direct effect exerted by Ia afferents, terminating monosynaptically on homonymous α-MNs and on their synergists [11, 12], these afferents activate also the number of spinal interneurons, affecting MN activity indirectly [13]. Low-threshold muscle afferents terminate on the spinal excitatory and inhibitory Ia interneurons, acting in concert with other peripheral, spinal and supraspinal inputs converging on these interneurons [13, 14]. Therefore, chronic stimulation addressed to Ia fibers in the tibial nerve might not only reinforce Ia glutamatergic input to the extensor MNs innervated by this nerve branch, but also exert widespread effect through spinal interneurons including those terminating on the cholinergic V0_c_ group.

Cholinergic C-terminals apposing soma of α- MNs originate from V0_c_—interneurons. Acting via muscarinic m2 receptors, acetylcholine released from C-terminals modulates the excitability of α- MNs causing its increase [15, 16]. V0_c_—interneurons receive among others, indirect input from sensory afferents but its modality remains unknown [17]. They respond to spinalization with profoundly reduced cholinergic innervation of α-MNs of the ankle extensor (soleus—Sol) but not of the flexor (tibialis anterior—TA) muscles [18]. Partial compensation of this deficit, by means of treadmill locomotor training of spinal animals, indicates that V0_c_-interneurons are sensitive to proprioceptive stimulation. It is possible, that low-threshold electrical stimulation addressed to Ia afferents of Sol muscle will activate also interneurons exerting excitatory effects on V0_c_—interneurons and indirectly affect the cholinergic input to α-MNs of the ankle extensors.

To verify these two hypotheses, both groups of synaptic terminals apposing cell bodies of α-MNs were identified by immunofluorescence (IF), using antibodies against vesicular transporters of glutamate (VGLUT1) and acetylcholine (VAChT) [15]. Ia boutons terminating monosynaptically on α-MNs are known to use VGLUT1 [13, 19–23] (for review see Alvarez, Bullinger [24]). We made an assumption, that Ia proprioceptive inputs on the lumbar α-motoneurons in the rat are the only ones which use VGLUT1 because corticospinal tract which contains VGLUT1 in majority of axons (96% acc. to [25]) does not form monosynaptic inputs on these motoneurons [26] and the other descending excitatory pathways use VGLUT2 [25]. To examine whether electrical stimulation of low-threshold proprioceptive afferents leads to a change of VGLUT1 and VAChT content in terminals, that may regulate quantal content and size and thus change frequency of synaptic vesicle release and synaptic strength [27–30], we carried out an analysis of VGLUT1 IF and VAChT IF intensity, making an assumption that it reflects transporter content in synaptic vesicles present in terminals.

Here we show that one week of continuous burst stimulation of low-threshold proprioceptive input to α-MNs innervating the ankle extensor muscles was effective not only in enrichment of their glutamatergic but also cholinergic innervation by increasing the number and aggregate volume of terminals. Moreover, it possibly led to an increase in the amount of glutamate and acetylcholine loaded to synaptic vesicles, suggesting that not only VGLUT1 glutamatergic but also VAChT cholinergic terminals respond to low-threshold stimulation with terminal growth and increased efficiency of neurotransmitter release.

## Materials and Methods

### Animals

The experiments were carried out on 12 adult male Wistar rats weighting 230–320 g at the beginning of the experiment. The animals were divided into two groups: (1) control, without electrode implantation (N = 6) and (2) experimental, subjected to bilateral implantation of the electrodes into Sol muscles and nerve cuff electrodes on the tibial nerve, and to unilateral electrical stimulation of low-threshold muscle afferents in that nerve (N = 6). Both groups of rats received bilateral intramuscular tracer injections to the LG. The animals were bred in the animal house at the Nencki Institute, Warsaw, Poland. They were given free access to water and pellet food and were housed under standard humidity and temperature conditions on a 12 h light/dark cycle.

Experimental protocols involving animals, their surgery and care were approved by the First Local Ethics Committee in Warsaw (no 535/2014) and were in compliance with the guidelines of the European Community Council Directive 2010/63/UE of 22 September 2010 on the protection of animals used for scientific purposes.

### Retrograde labeling of motoneurons, implantation of electrodes and postsurgery care

A surgery necessary for intramuscular injections of tracer to label MNs and implantation of the electrodes were performed during the same session. The animals were given subcutaneous (s.c.) injection of Butomidor (Butorfanolum, Richter Pharma, 1.5 mg/300 g b.w.) as a premedication and then were anesthetized with isoflurane (Aerrane, Baxter, 1–2.5% in oxygen) via a facemask. The skin overlying LG muscle was shaved, disinfected with 3% hydrogen peroxide, and cut. Fluorescent neurotracer True Blue (TB) (15 μl of 1% aqueous solution of TB Chloride T0695, Sigma-Aldrich, St. Louis, MO, USA) was injected to LG muscles by means of Hamilton microsyringe with attached 22-gauge needle which was gradually advanced from the distal toward the proximal end of the muscle belly. During 5 min delivery of TB the needle was slowly withdrawn; after the injection was completed the needle was left in the muscle for at least 3 min in order to avoid leakage of the dye. The injection site was cleaned after retraction of the needle and the skin was sutured.

**Fig 1:**
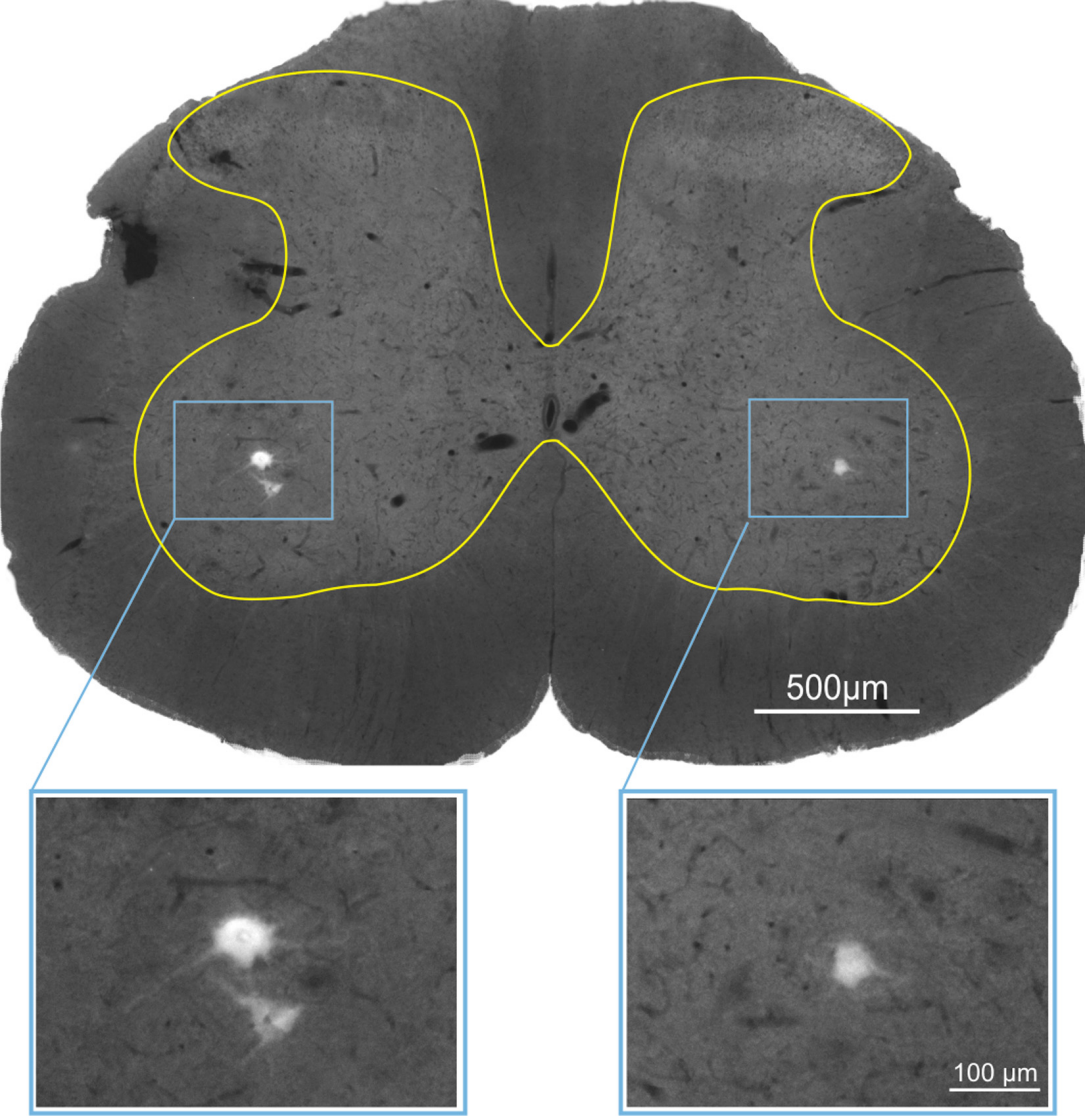
Example of typical location of α-motoneurons innervating LG muscle in L5 spinal segment. The spinal grey matter borderline is marked. Retrogradely labeled motoneurons (framed) are shown enlarged below. Motoneurons were labeled with True Blue fluorescence tracer injected into the LG muscle belly.

Labeled LG MNs were predominantly located in L5 segment, as expected, and their typical location in the spinal grey is shown in Fig 1.

Procedure and the surgery necessary for implantation of the electrodes were the same as those described in detail in our former article [10]. Briefly, a connector plug for the electrodes was sewn to the muscles and ligaments over the vertebrae. The electrodes were drawn subcutaneously, bilaterally, from the connector plug mounted at the back, to the Sol muscles and tibial nerves to be implanted. Bipolar cuff electrode was performed as described by Loeb and Gans [31] and implanted over the tibial nerve which was separated from the common peroneal and sural branches in the popliteal fossa. Low-threshold stimulation of the tibial nerve produced a compound muscle action potential, which was recorded in the Sol muscle by means of a pair of Teflon-coated fine-wire electrodes with 1.5 mm final bare spaced apart by about 5 mm. To avoid leakage of the dye from the Sol muscle with electrodes implanted, the dye was injected to the LG muscle, a close synergist of Sol, which is also innervated by tibial nerve branches and subjected to electrical stimulation via cuff electrode.

After the surgery, Baytril (Enrofloxacinum, 5 mg/kg, s.c., Bayer) was administered over five consecutive days to prevent infection. An analgesic Tolfedine (Tolfenamic acid 4%, 4 mg/kg, s.c., Vetoquinol S.A.) was given during the first three postoperative days. Immediately after the surgery, the rats were placed in warm cages, covered with blankets and inspected until fully awaken. Thereafter plastic collars were put on each animal to protect their wounds from licking, and rats were returned to individual cages with full access to food and water.

### Behavioral training

To reduce the effects of the behavioral context and ongoing motor activity on the recorded H-reflexes the animals were accustomed to being restrained in the apparatus which limited their body movements. Therefore, approximately three weeks before the stimulation experiment was initiated, the animals started to be accustomed to sitting in the immobilizing apparatus until they reached 20 min criterion [10].

### Stimulation and H-reflex recording

**Fig 2:**
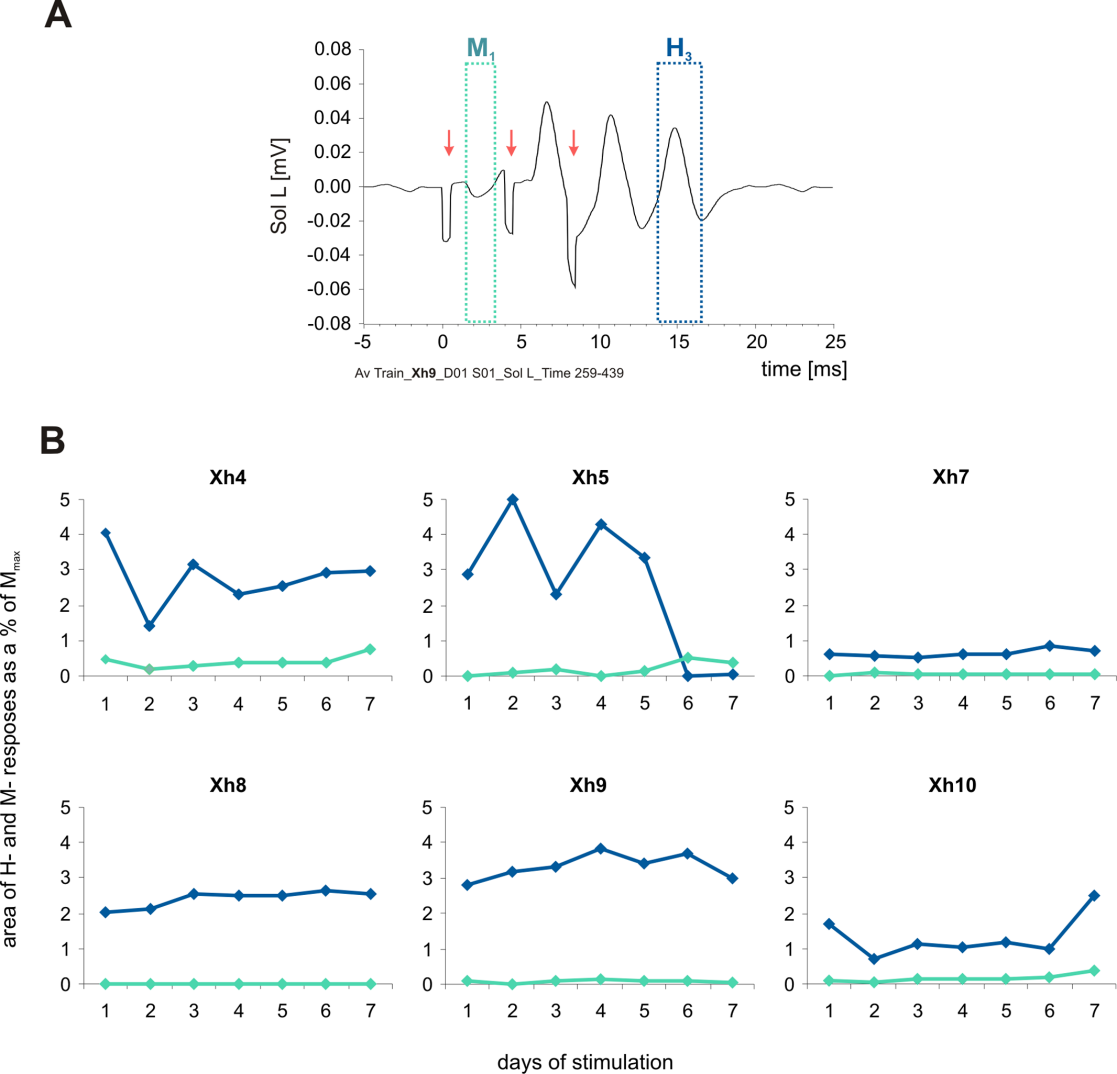
The direct M_1_ responses and H_3_-reflexes recorded in the rat soleus muscle during stimulation sessions of low-threshold proprioceptive fibers in the tibial nerve. **A.** Examples of the raw data averaged after 7200 burst repetitions. Arrows indicate stimulus artifacts. **B**. The mean areas of the H_3_-reflexes (blue line) and M_1_-responses (green line) during consecutive days of stimulation in six rats. Four 3 min samples daily were taken for the analysis: at the beginning and at the end of the first and fourth stimulation sessions. The data are expressed as a percentage of M_max_ values for individual animals.

The monosynaptic H-reflex was elicited by electrical stimulation addressed predominantly to low-threshold muscle afferents (Ia group) in the tibial nerve and recorded as a compound muscle action potential in the Sol muscle as described in detail in our former article [10]. Briefly, the tibial nerve was stimulated unilaterally and the contralateral side, with implanted electrodes, served as a sham-stimulated side. The experiments started with testing an individual threshold current values producing the H-reflex and/or threshold M-responses to single stimuli at 0.3 Hz (see [10]). Once the thresholds were established we have started stimulation session monitoring the size of the M1-response. We monitored both the H- reflexes and M-responses and corrected the current, if necessary. Continuous bursts of three pulses (pulse width = 200 μs with 4 ms inter-pulse interval) were delivered every 25 ms to the tibial nerve in four 20 min sessions daily for seven consecutive days [32]. After each session the animals rested at least 1 hour in their home cages and were rewarded with corn cookie. The strength of stimulation was established at the threshold of excitation of the motor fibers, which is higher than the one activating Ia afferents. Therefore, it elicited a fair H-reflex, since the majority of Ia fibers are already excited when the direct motor response (M) is at its threshold [33], Czarkowska-Bauch (unpublished). Only the M-responses elicited by the first out of three pulses in the burst were strictly controlled and kept at its threshold as these responses were not contaminated by subsequent H-reflexes (see Fig 2A).

### Data acquisition and analysis of electrophysiological data

Data acquisition and analysis of electrophysiological data have been described in detail in our former article [10]. Briefly, after amplification the analog EMG signals were fed to a CED Micro 1401 ^mk II^ interface (Cambridge Electronic Design Ltd, UK), digitized and fed to a PC. Raw EMG activity was also monitored throughout the experiment on the oscilloscopes. A Spike 2 (Cambridge Electronic Design Ltd, UK) based script was used to measure the latency, peak-to-peak amplitude and the area of the H-reflex and M-response and to average these data. These results were expressed as a percentage of M_max_ elicited by single-pulse stimuli and collected in each animal after last session on the 6^th^ day of stimulation.

### Tissue processing

Within the 1.5 h after the last training session, the animals were anaesthetized with lethal dose of sodium pentobarbital (80 mg ⁄ kg, i.p.) and transcardially perfused with 250 ml 0.01M phosphate-buffered saline, pH 7.4, at room temperature (RT) and, subsequently, with 450 ml ice-cold 4% paraformaldehyde. Spinal cords were removed from the vertebral columns and were postfixed in the same fixative for 1 hour at 4°C. Tissue was then cryoprotected stepwise in 10%, 20% and 30% sucrose in 0.1M PB at 4°C and stored at 4°C until use. The L1-L5 spinal cord segments were surrounded by Jung tissue-freezing medium (Leica) and frozen in cryostat at -70°C. After tissue blocks were placed on tissue holders, the sham-stimulated side was marked with thin needle. Transverse 25μm thick sections were cut on the cryostat at -20°C, collected free-floating in anti-freeze cryoprotectant solution (300 ml glycerol, 500 ml 0.05 M PB pH 7.4, supplemented with 150 g sucrose and 90 mg thimerosal) and kept at -18°C until further processing.

### Chemicals and reagents

We used primary mouse monoclonal antibody against vesicular glutamate transporter 1—VGLUT1 (Cat. No. 135 511) and guinea pig polyclonal anti-synaptophysin antibody (Cat. No. 101 004), both from Synaptic Systems GmbH, Göttingen, Germany; rabbit polyclonal antibody against vesicular acetylcholine transporter -VAChT from Chemicon (V5387, Merck-Millipore, Billerica, USA). Secondary antibodies conjugated with Alexa Fluor 647 and DyLight 594 were from Jackson Immuno-Research Lab Inc. West Grove, USA, and that conjugated with Alexa Fluor 488 was from Invitrogen, Carlsbad, USA. All other chemicals and reagents were from Merck-Millipore or POCh, Poland.

### Immunofluorescent labeling of synaptophysin and vesicular glutamatergic and cholinergic transporters

IF triple-labeling was carried out in one experimental session to assure identical conditions of tissue processing and staining. The free-floating sections (6–14 sections per animal, the number depended on the presence of bilateral, well-defined, strong signal of TB in MNs) were washed in PBST and incubated with a solution of 2.5% normal goat serum (NGS) and 2.5% normal donkey serum (NDS) in PBST for 30 min at RT, in order to reduce the non-specific staining. Next, sections were incubated overnight at 4°C with rabbit anti-VAChT (1:1000) combined with mouse anti-VGLUT1 (1:500) and guinea pig anti-synaptophysin antibodies (1:1000), diluted with PBST. Then sections were washed in PBST, prior to 1h incubation at RT with the respective secondary antibodies: Alexa Fluor 647 donkey anti-mouse (1:400), Alexa Fluor 488 goat anti-rabbit (1:500) and DyLight 594 goat anti-guinea pig (1:200). After several washes, the sections were mounted onto glass slides, air-dried, mounted in Mowiol medium, coverslipped and kept in the dark at 4°C until analysis. The regular staining procedure was preceded by the trial with single and triple labeling to test whether simultaneous triple reaction does not cause impaired or non-specific antibody binding. Negative controls were obtained by omitting the primary antibodies.

### Image acquisition with confocal microscope, deconvolution and selection criteria

The labeled sections were examined with confocal inverted microscope LSM 780 (Carl Zeiss, Jena, Germany). Z-stacks of images of labeled MNs and terminals apposing their perikarya consisted of digital slices collected at 0.21 μm intervals with a pixel size of 0.073 using PL APO 63x / 1.4 Oil DIC oil-immersion objective. Images were collected at constant exposure parameters for each of four channels with IF labeling for TB, VGLUT1, VAChT, and synaptophysin.

**Fig 3:**
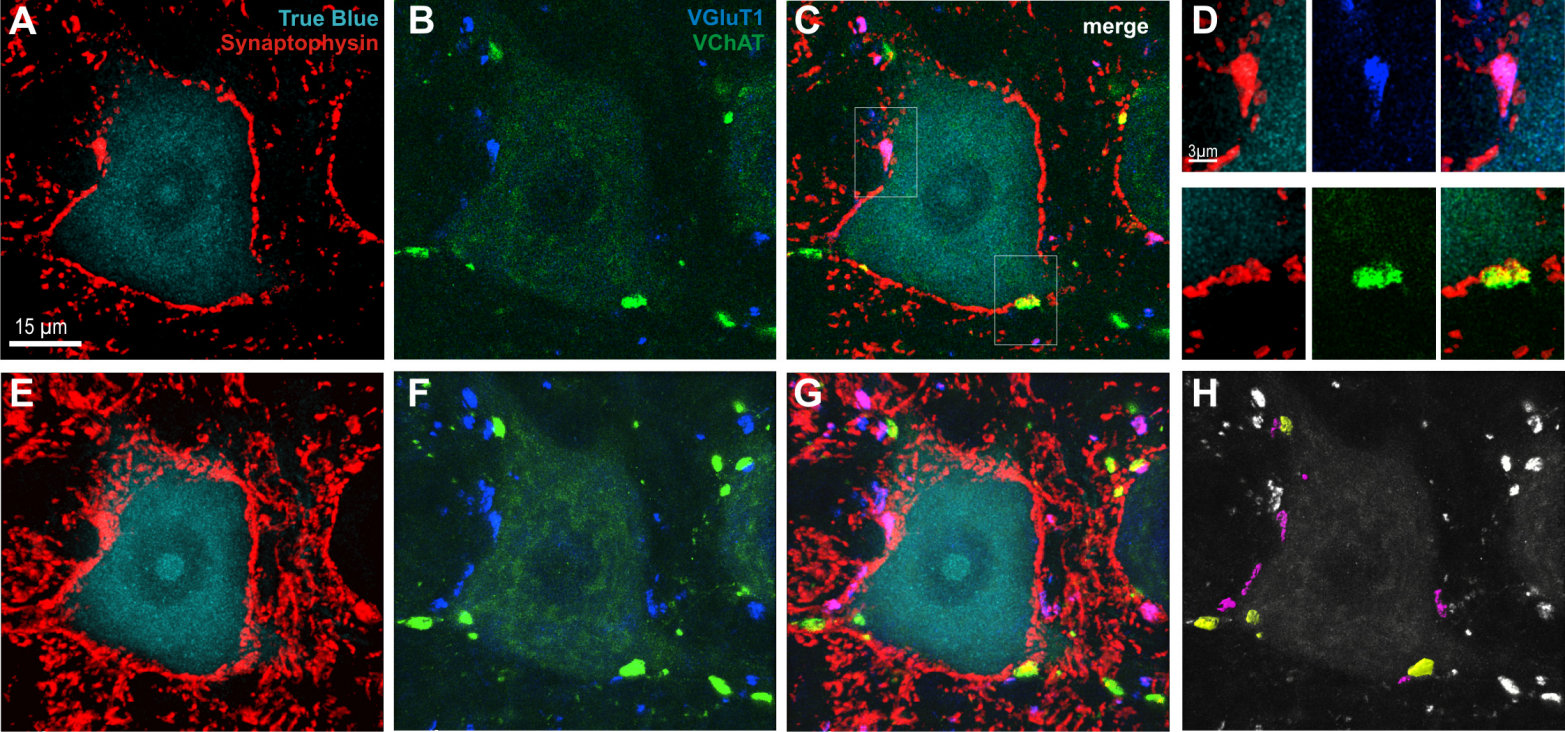
Identification of glutamatergic (VGLUT1; indigo) and cholinergic (VAChT; green) terminals abutting on LG α-motoneuron (α-MN) labeled by means of immunofluorescence (IF). LG MN perikaryon and proximal parts of dendrites were identified with True Blue (turquoise; A, C, E and G) and all synaptic terminals apposing LG α-MN were identified by synaptophysin IF (red). A-C present single optical section (0.21 μm thick); framed areas on C (merge of A-B) are shown with higher magnification in D to demonstrate contiguity of VAChT or VGLUT1 varicosities with the edge of the α-MN perikaryon. E-H are stacks of 20 optical sections of the same α-MN to show 3D reconstruction of the glutamatergic (indigo) and cholinergic (green) terminals among all synaptic terminals (synaptophysin) abutting on LG α- MN. G–merge of E and F. H—glutamatergic (magenta) and cholinergic (yellow) terminals shown to contact α-MN surface were accepted for quantification while the other (white) terminals, which did not fulfill these criteria, were not analyzed.

Z-stacks of images were subjected to deconvolution procedure using Huygens Professional (Scientific Volume Imaging, Hilversum, Netherlands) to reduce the image distortion arising from light scattering. Based on random set of images an experimentally blind rater determined the number of iterations for the convolution algorithm to yield maximal resolution for each antibody (40 iterations for all channels). The level of background and signal to noise ratio was selected for each channel independently. Only profiles of LG α-MN containing TB tracer and visible cell nuclei together with VAChT and VGLUT1 IF terminals abutted on the VAChT IF cell bodies and initial segments of proximal dendrites of these MNs were taken into account, outlined and evaluated. Whether there is a contiguity of glutamatergic and cholinergic varicosities to the cell body or proximal part of a dendrite and their overlap with synaptophysin was examined on single optical scans (see Fig 3A–3D). A segment of a dendrite within 50 microns from the soma has been taken as a proximal dendrite, after a classical study by [34] who showed, that a similar number of boutons contact the soma and proximal dendrites in FF, FR and S motoneurons, in contrast to dendritic segments located more distally.

The number, volume and intensity of VGLUT1 and VAChT IF boutons apposing perikarya of LG α-MNs were measured by means of Imaris 7.0 software (Bitplane, South Windsor, CT, USA) on 3D reconstructions based on acquired confocal images.

To create a detailed, three-dimensional surface rendering of the IF signal of synaptic terminals we used two automatic thresholding functions of the software. The threshold values were automatically computed using algorithms providing the best fit based on [35]. The choice of the algorithm depended on the characteristics of IF distribution within the whole image.”Background subtraction" (local contrast) thresholding method for VGLUT1 IF was applied. For VAChT IF an "absolute intensity" function was chosen, because the intensity of VAChT IF in C-boutons was much stronger than that in α-MN perikarya (see Fig 3) enabling unequivocally differentiate those terminals. The level of detail capturing on surfaces of selected three-dimensional objects was set at 0.2 μm, as this provides high accuracy and limits a noise level.

The volume (in μm^3^) and mean IF intensity (arbitrary units) values for selected objects were provided by the software. We used “volume filter” for synaptic terminals. The criteria for the volume of glutamatergic terminals (objects larger than 1 μm^3^) and cholinergic terminals (objects larger than 5 μm^3^) for the intact animals were adopted from the studies by [36, 37] and used for VAChT measurements in our previous study [18]. The numerical data were exported to Excel for further analysis. The mean and aggregate volume of all terminals was calculated.

The number of examined LG α-MNs and the number of VGLUT1 IF and VAChT IF synaptic terminals abutting on these MNs are shown in the Table 1.

**Table 1:** The number of TB-identified LG α-MNs subjected to the analysis of VGLUT1- and VAChT IF synaptic terminals apposing their cell bodies. (N)—the number of animals per group. The numbers of analyzed synaptic terminals are shown in square brackets.

Number of LG α-motoneurons and synaptic terminals:	Control	Sham-stimulated	Stimulated
	(N = 6)	(N = 6)	
VGLUT1 IF	17 [317]	31 [490]	33 [610]
VAChT IF	22 [375]	31 [495]	33 [586]

### Quantification of the results

The mean number of optical images (collected at 0.21 μm Z-intervals) per α-MN was 64 (± 12; SD). Therefore, the Z-stack of digital images allowed for reconstruction of 30–50% of the whole surface of MN soma used to count the number and measure the volume of individual synaptic terminals on its surface. The calculation of α-MNs soma surface under analysis was based upon labeling of somatic cytoplasm with TB and VAChT, and verified with synaptophysin IF forming clear outline surrounding the plasma membrane and defining the contour of soma on every fourth scan (see Fig 2A). The obtained contours were multiplied by 4 and by thickness of a single optical slice and summed up. The mean of lateral surface of analyzed fragments of the α-MNs soma was 4772 μm^2^ (±1214; SD) and was balanced between groups. In order to normalize the results, the density of synaptic boutons was expressed as the number of boutons abutted on 3000 μm^2^ of the MN soma surface, i.e. the same fragment of the MN soma, which we referred to in our previous study [18].

The figures were assembled using COREL DRAW X5 software.

### Statistical analysis

Not all sets of data met criteria of parametric tests (verified with *Shapiro-Wilk normality test* and *Leven homogeneity of variance test*) therefore we applied statistical analysis with the use of non-parametric tests. The *Wilcoxon and Mann-Whitney U tests* were used for comparisons of related samples and independent samples, respectively. STATISTICA 10 software (StatSoft. Inc. Tulsa, OK, USA) was used for the data analysis.

## Results

The electrophysiological data obtained from the animals, which were used in this study, were analyzed and described in our previous article [10]. Briefly, the latencies of H-reflexes in the soleus muscle were approximately 6 ms whereas those of M-responses were approximately 2 ms. Setting the strength of stimulation at the threshold for eliciting M-responses which is higher than that activating Ia afferents, and continuous monitoring the M-responses enabled activation of majority of low-threshold muscle afferents [33]. Delivering of continuous bursts of stimuli created stable conditions for eliciting H-reflexes with relatively low variability of the responses.

Example of the raw data after averaging is shown in Fig 2A. Only M_1_ response and H_3_ reflex (delineated) were analyzed as they were not “contaminated” by accompanying M or H responses recruited after 2nd and shortly after 3rd stimulus. M_1_ and H_3_ responses were subjected to further analysis and expressed as a percentage of M_max_ during consecutive days of stimulation (Fig 2B). The size of threshold M_1_ responses was stable throughout the experiment. The size of H-reflexes was also stable in four out of six animals whereas in two other rats it did not change consistently in time of the stimulation (in the rat Xh5 technical problems appeared after five days of stimulation causing loss of H-reflexes). Analysis of the size of H_3_ reflexes shows that relatively small percentage of α-MNs in the pool of Sol was activated by the third stimulus of the burst. Considering that aspect one should take into account the H_1_- and H_2_-reflexes, although not quantified, contribute also to the excitation of that pool.

### Chronic stimulation of low-threshold proprioceptive afferents enriches glutamatergic innervation of LG α-motoneurons with small boutons

The proprioceptive Ia afferents, activated by electrical stimulation of the tibial nerve in the H-reflex loop, are known to terminate monosynaptically on the soma and dendrites of homonymous MNs with terminals carrying type 1 vesicular transporter of glutamate (VGLUT1; see Fig 3) [19, 21–23].

**Fig 4:**
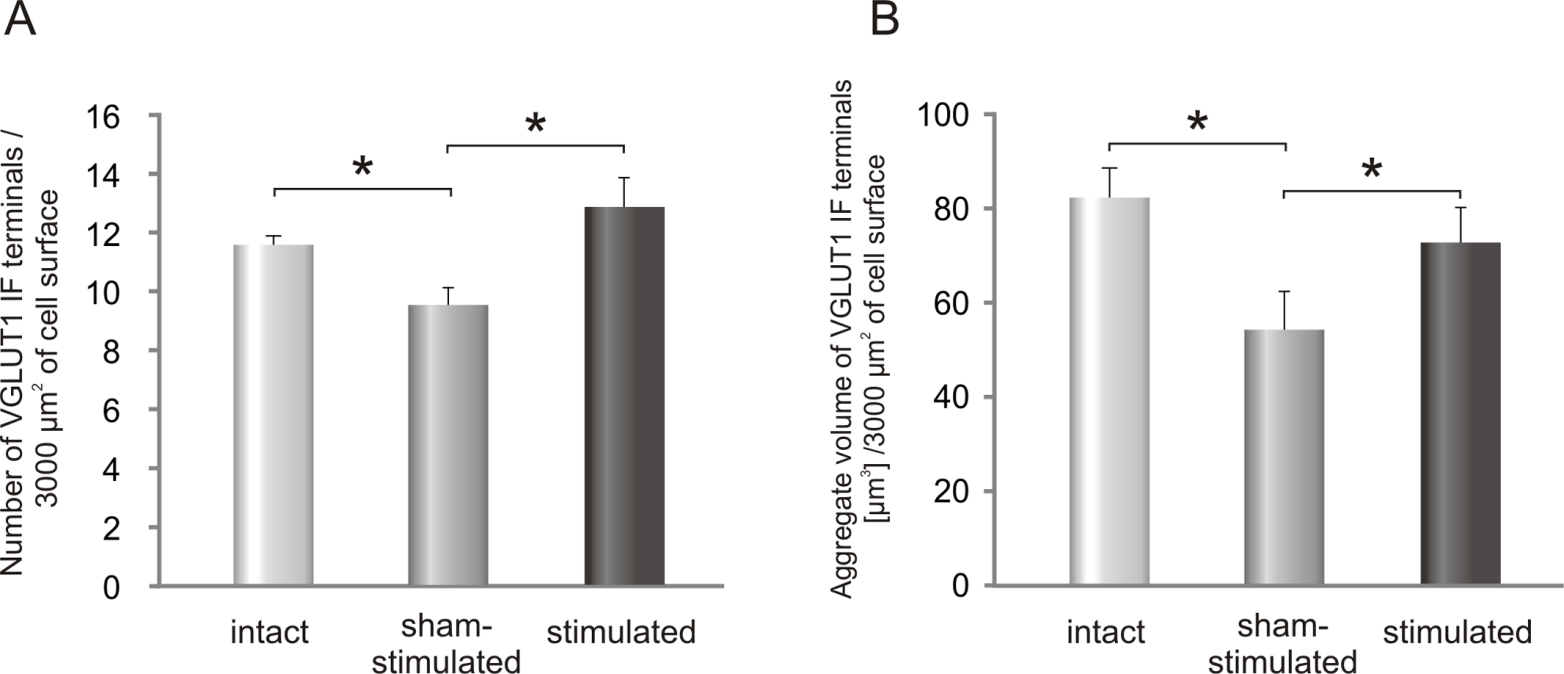
**Changes in the number (A) and in the aggregate volume (B) of VGLUT1 IF synaptic terminals contacting LG α-MNs after seven days of stimulation of Ia fibers in the tibial nerve.** Data are reported as mean +/- SEM. Both the number and aggregate volume of VGLUT1 terminals were increased on the stimulated comparing to sham-stimulated side (*p = 0.03 and *p< 0.05, respectively, *Wilcoxon* test).

To reveal the effect of seven days of low-threshold proprioceptive stimulation on glutamatergic innervation of LG α-MNs, the density and morphology of glutamatergic VGLUT1 IF terminals apposing to the soma and the most proximal part of the dendrite (up to 10 μm) were evaluated. The stimulated side was compared with the sham-stimulated side of the lumbar spinal cord and with control group (Table 1). Fig 4A shows that the number of VGLUT1 IF terminals contacting perikarya of LG α-MN increased after stimulation by about 35% comparing to the sham-stimulated side (mean ± SEM /3000 μm^2^; *p< 0.03, *Wilcoxon test*). Surprisingly, the number of VGLUT1 IF boutons was decreased in the sham-stimulated side comparing to the control (*p<0.03, *Mann-Whitney U test*). Whereas the mean volume of terminals was decreased in the implanted animals by 20% (sham-stimulated: 5.6 ± 0.7μm^3^; stimulated: 5.7 ± 0.3 μm^3^; controls: 7.2 ± 0.5μm^3^; means ± SEM), the aggregate volume of these terminals/ 3000 μm^2^ of MN soma surface, which was lower by 34% in the sham-operated side (*p = 0.03, *Mann-Whitney U test*) than in the control group (Fig 4B), showed an increase after stimulation by 35% (*p<0.05, *Wilcoxon test*). Thus stimulation largely compensated a decrease in the number and aggregate volume of VGLUT1 terminals that occurred after implantation (Fig 4B).

### Stimulation causes a shift in distribution of VGLUT1 IF boutons towards smaller objects

**Fig 5:**
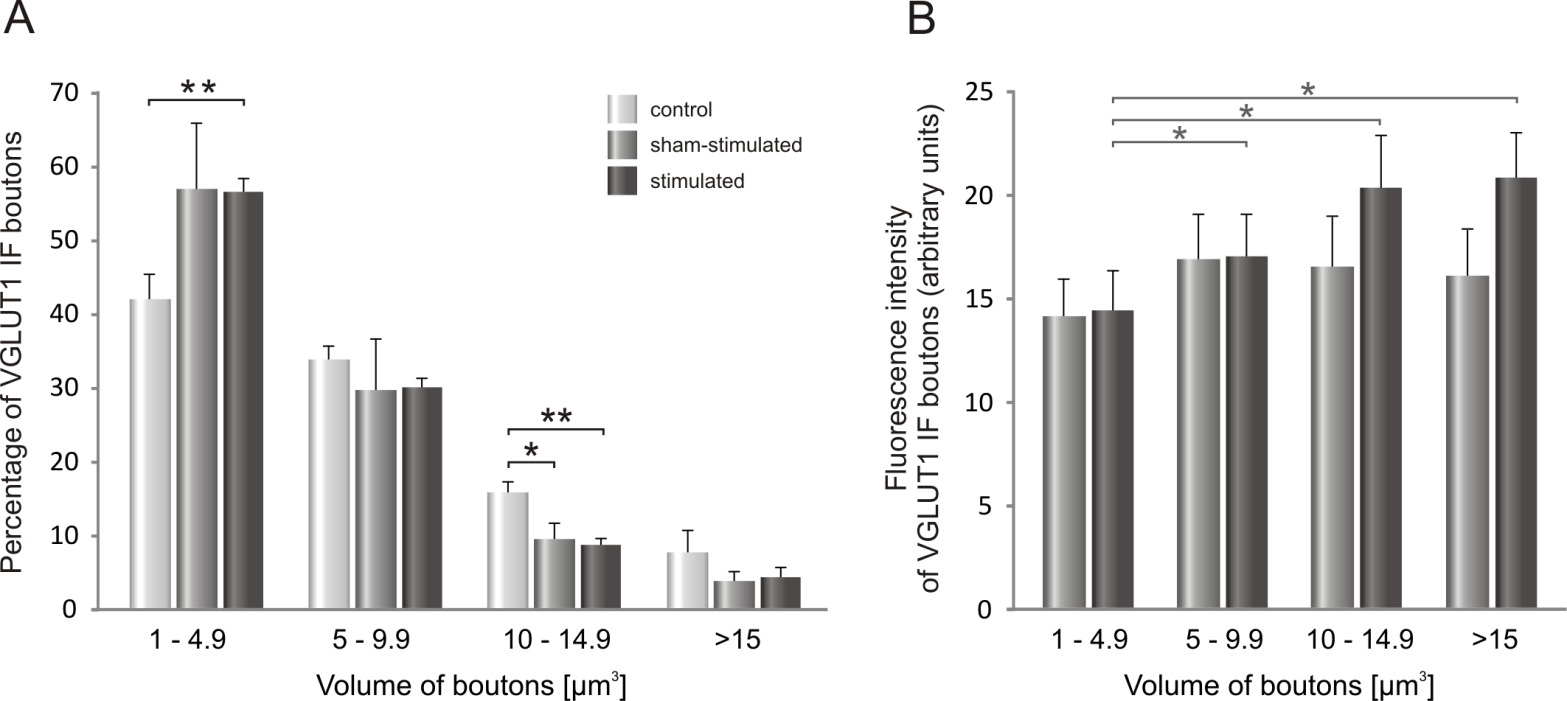
**A.** The distribution of VGLUT1 IF boutons of different volume apposing to LG α-motoneurons after seven days of low-threshold stimulation of proprioceptive fibers in the tibial nerve. **B.** Intensity of VGLUT1 IF signal in boutons apposing to LG α-MNs plotted in function of their volume on the sham-stimulated and electrically-stimulated side. Data are reported as mean +/- SEM. Stimulation of Ia fibers resulted in an increased number of VGLUT1 IF terminals of the smallest size at the expense of the largest (volume > 10 μm^3^) terminals (*p<0.03, **p<0.005, *Mann-Whitney U test*). An intensity of the signal tended to increase in the largest boutons.

A decrease in the mean volume of glutamatergic varicosities raised a question, whether this phenomenon concerns the entire population of VGLUT1 terminals of different sizes contacting LG MNs. To analyze possible shifts in volume of VGLUT1 varicosities on both sham- and stimulated side, we carried out analysis of frequency distribution of boutons of various volumes, distinguishing arbitrarily 4 classes of terminals (Fig 5A).

Poisson-like distribution of the VGLUT1 IF terminals apposing perikarya of LG α-MNs in function of their volume shows the highest frequency of the smallest boutons both in intact and stimulated animals (Fig 5A). A significant increase of the number of VGLUT1 terminals was observed in that group of boutons, ranging between 1 and 4.9 μm^3^, on the stimulated side (Fig 5A, **p<0.003, *Mann-Whitney U test*). On the contrary, the number of big boutons ranging between 10 and 14.9 μm^3^ was decreased in stimulated compared to control rats (on the sham- stimulated side *p<0.03; on the stimulated side **p<0.005; *Mann-Whitney U test*). Combined data on changes in the number and volume of VGLUT1 terminals may indicate that seven days of stimulation of low-threshold proprioceptive afferents is sufficient to cause a formation of new glutamatergic terminals, which are characterized by their small volume at this stage. Newly formed VGLUT1 IF terminals may not only be smaller but also functionally less efficient than the other, containing less amount of the vesicular transporter of glutamate than larger boutons [27, 29].

To examine the latter possibility, we measured an intensity of VGLUT1 staining within synaptic terminals abutting on LG MNs. This analysis showed that in control animals a gradient of intensity occurs with gradual increase of signal density in function of terminal volume. VGLUT1 staining intensity showed a tendency to be higher by 25% in the biggest (p = 0.11, *Wilcoxon test)*, and was higher by 31% in the medium (p<0.03, *Wilcoxon test*) and by 18% in the small terminals (p<0.05, *Wilcoxon test*), comparing to the smallest ones. In the implanted rats sham operation suppressed a gradient whereas after stimulation the gradient appeared similarly as in control group, with an increase of VGLUT1 IF signal by 44% in the biggest (p<0.03, *Wilcoxon test*), 41% in the medium (p<0.03; *Wilcoxon test*) and 18% in the small, as compared to the smallest terminals (Fig 5B). Our results provide no evidence for increased VGLUT1 density at small boutons after chronic stimulation, being in line with our hypothesis of their functional immaturity.

### The effect of chronic stimulation of low-threshold proprioceptive afferents in the tibial nerve on cholinergic inputs to LG α-motoneurons

**Fig 6:**
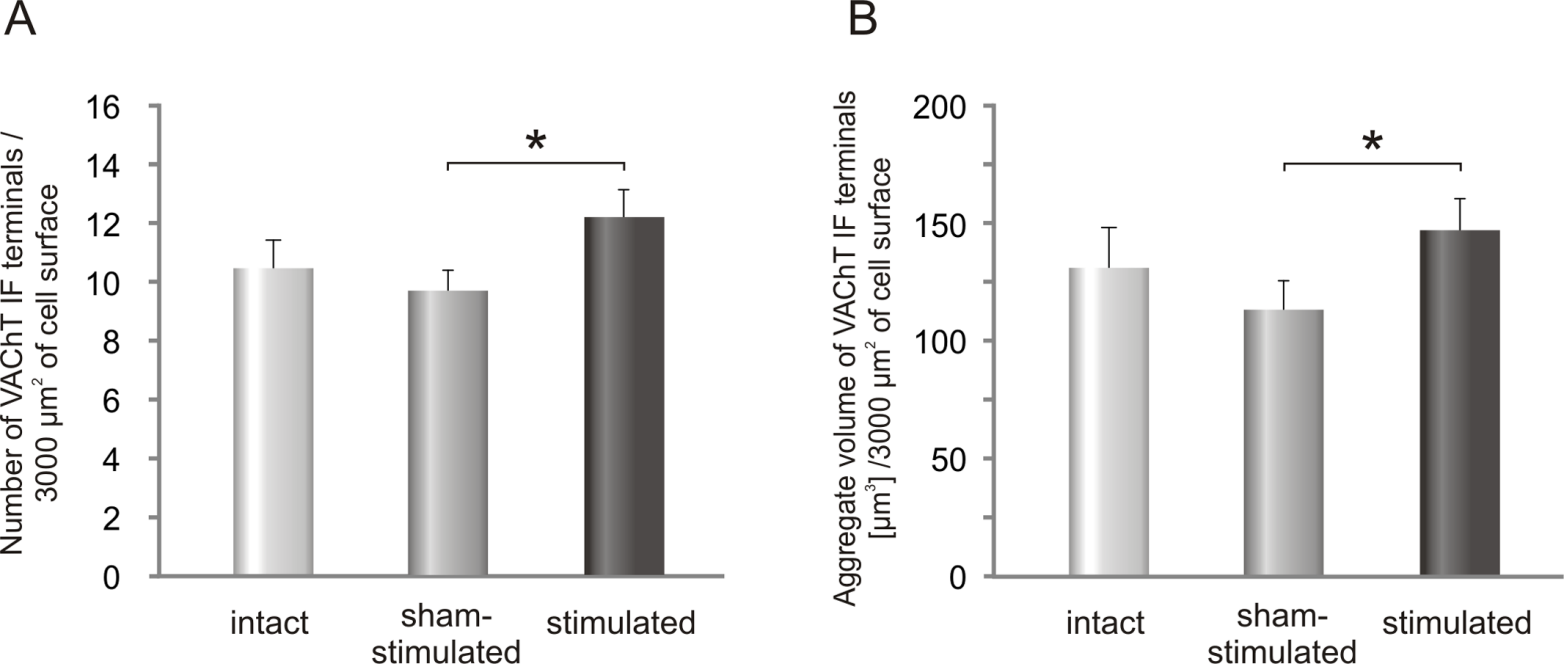
**The effect of unilateral stimulation of low-threshold proprioceptive afferents in the tibial nerve on the number (A) and aggregate volume (B) of VAChT IF terminals contacting LG α-MNs.** Data are reported as mean+/- SEM. The number of VAChT IF terminals increased by 26% after stimulation compared to sham-stimulated side (*p< 0.03, *Wilcoxon test*).

Cholinergic VAChT IF boutons abutting on comparable number of TB-identified LG α-MNs localized on the stimulated- and on the sham-stimulated side were analyzed and compared with those in control animals (Table 1). The number of VAChT IF terminals contacting the soma of LG α-MNs and the most proximal part of the dendrite (up to 10 μm) increased after stimulation by 26% comparing to sham-stimulated side (Fig 6A) (*p< 0.03, *Wilcoxon test*). The number of VAChT IF terminals apposing LG α-MNs on the sham-stimulated side was close to that in controls (Fig 6A).

**Fig 7:**
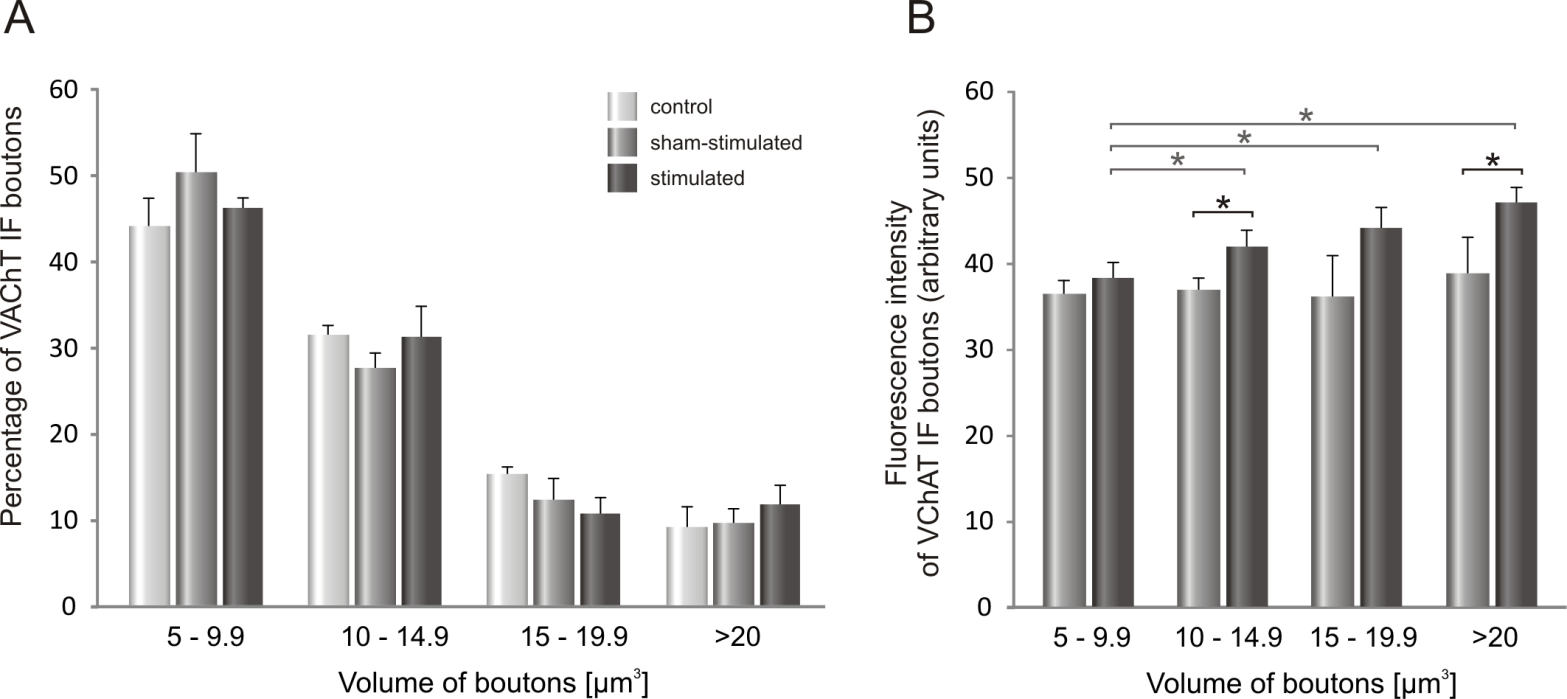
**A.** The distribution of VAChT IF boutons of different volume apposing LG α-motoneurons after seven days of stimulation of low-threshold proprioceptive fibers in the tibial nerve. **B**. Intensity of VAChT IF signal in boutons apposing LG α-MNs plotted in function of their volume on the sham- and stimulated- side. Data are reported as mean +/- SEM. Stimulation of Ia fibers resulted in an increased intensity of signal in VAChT IF boutons (* p<0.05, *Wilcoxon test*).

The mean volume of VAChT IF terminals was not affected by stimulation (sham-stimulated: 11.7 ± 0.6 μm^3^; stimulated: 12.3 ± 0.3 μm^3^; controls: 12.9 ± 1.0 μm^3^; means ± SEM). However, the aggregate volume of VAChT terminals (Fig 6B), which did not change significantly on the sham-stimulated side, was increased by 30% after stimulation (*p<0.03, *Wilcoxon test*). As shown on Fig 7A, the percentage of the smallest boutons did not change after stimulation, comparing to control ones.

Interestingly, by comparing the distribution patterns of cholinergic and glutamatergic terminals of different volumes (Figs 5A and 7A), we have found that both in control and stimulated animals proportions of distinguished subclasses in populations of VGLUT1and VAChT terminals were comparable, with the most numerous subclass of the smallest boutons (approximately 40%).

Analysis of intensity of VAChT staining within C- terminals abutting on LG MNs showed that in control animals a gradient of intensity occurs. It is less expressed than that in VGLUT1 terminals. VAChT staining intensity showed a tendency to be higher by 22% in the biggest (p = 0.11, *Wilcoxon test*), and was higher by 18% in the medium (p<0.03, *Wilcoxon test*) and in the small terminals (p<0.05, *Wilcoxon test*), comparing to the smallest ones. In the implanted rats sham operation suppressed a gradient whereas after stimulation the gradient reappeared. VAChT IF signal was higher by 23% in the biggest (p<0.03, Wilcoxon test), by 15% in the medium (p<0.05; *Wilcoxon test*) and by 10% in small terminals (p<0.03; *Wilcoxon test*), as compared to the smallest ones (Fig 7B). After stimulation VAChT IF signal intensity tended to increase in C-terminals, irrespectively of their volume, with significant effects in classes: 10–14.9 and over 20 μm^3^ (*p<0.05, *Wilcoxon test*) (Fig 7B).

## Discussion

Our study shows that one week of continuous burst stimulation of proprioceptive input to LG α-motoneurons is effective in increasing the number and aggregate volume of their direct glutamatergic but also indirect cholinergic inputs.

To keep control over the low-threshold stimulation addressed predominantly to Ia muscle afferents we recorded the monosynaptic H-reflex and accompanying near-threshold direct motor response (M). It was established that when M-responses are at their threshold we can elicit fair H-reflexes, since the majority of Ia fibers are already excited [10, 33]. However, because the threshold of excitation of the Golgi tendon afferents is close to that of low-threshold muscle spindle afferents (Ia) and we are not able to control that component in awake animals, we use “low-threshold proprioceptive afferents” term instead of “Ia afferents”.

Analysis of the size of H_3_ reflexes shows that relatively small percentage of α-MNs in the pool of Sol is activated by the third stimulus of the burst. However, H_1_- and H_2_-reflexes, which were not quantitatively analyzed, also contribute to the excitation of that pool. Another important factor determining the responses of the MNs to the stimulation is the functional state of the MN pool under study. In awake animals it is modulated by ongoing motor activity, despite the animals are well accustomed to the experimental situation [38].

### Does chronic stimulation of low-threshold proprioceptive afferent fibers in the tibial nerve affect equally effectively LG and Sol motoneurons?

To control a stimulation of low-threshold proprioceptive fibers in the tibial nerve addressed to muscle afferents originating from the Sol muscle receptors we monitored the H-reflexes and the direct M-responses recorded in this muscle. The synaptic effects of this stimulation on innervation of α-MNs were, however, evaluated in heteronymous LG MNs, Sol synergist, identified by means of retrograde tracer injected to LG muscle. This experimental arrangement was chosen to avoid an uncontrolled leakage of the dye which, when injected to implanted Sol muscle, might affect tracing efficiency and data reproducibility. The assumption which allowed us to use that stimulation paradigm was based on well documented data [11, 39–41], that conduction velocity and the threshold of activation of Ia afferents of Sol and gastrocnemius (GS) muscles in cats are similar. If so, low-threshold stimulation of the tibial nerve, sufficient to elicit H-reflexes in Sol muscle, should effectively activate Ia fibers encapsulating also muscle spindle afferents of LG muscle. When the thresholds of H-reflexes are compared in humans the current sufficient to elicit the H-reflex in Sol muscle is slightly lower than in the medial gastrocnemius (MG) or LG muscles and that of M-responses are also similar [42, 43]. However, it cannot be excluded that the same stimulus strength will favor activation of Sol comparing to LG MNs. It has been shown that the same stimulus produces H-reflexes of higher amplitude in Sol than in LG or MG muscles [43, 44] suggesting its greater impact on Sol- than on LG MNs. This effect might originate from approximately twice greater number of muscle spindle receptors in the Sol than in GS muscles found both in rats [44] and humans [42, 45].

Importantly, LG MNs receive not only homonymous monosynaptic Ia input but they are also supplied by Ia heteronymous input originating from Sol muscle [11, 12, 46]. Therefore, the net Ia excitatory effects observed in LG MNs might be additive.

It has to be taken into consideration that lower threshold of motor axons of fast LG MNs (Type F) than that of slow (Type S) Sol MNs, described by Munson and co-authors (1997) [47] in cats may characterize also rat LG Type F and Sol Type S motor axons. If so, also in our experimental model stimulation kept at the threshold of M-responses of Sol MNs could preferentially activate type F LG MNs.

Our stimulation paradigm has been focused on the reinforcement of low-threshold proprioceptive input to α-MNs under study but to control the strength of stimulation we have monitored a direct motor (M) response, which was kept at its threshold (see Fig 2). It is therefore possible that chronic stimulation of Ia and motor axons affects the synaptic input to the stimulated motoneurons not only via orthograde signaling but also retrograde influences originating from the muscles and thus both might affect electrical properties of MNs [47]. It is worth stressing that in experiments documenting the role of retrograde influences exerted by muscle on MNs the strength of chronic stimulation was much higher [47, 48] than in our experiments.

Neurotrophins secreted by the muscles were postulated to play a role here [47, 49]. Pronounced increase of NT-3, both in the L3-L6 spinal segments and in Sol muscle, observed by us after the same stimulation paradigm speaks in favor of that possibility [10].

### Susceptibility of the tibial nerve to implanted cuff electrode

Decreased number of VGLUT1 IF terminals on the sham-stimulated side comparing to that in control group suggests that the cuff electrodes implanted around the tibial nerves could cause some damage of the afferent fibers, although an internal diameter of the cuff electrode clearly exceeded that of the tibial nerve diameter. Susceptibility to the damage of peripheral nerves, which were enclosed over months in the cuff electrodes, was reported by several groups. Evidence of benign fiber loss was reported 4 months after surgery in cats based upon the measurement of conduction velocity of compound nerve action potentials [50]. Stein and co-authors observed a small reduction in the number of large diameter fibers in the nerve enclosed over 9 months in the cuff electrode in cats [51]. In rats, the long-term (3–10 months after surgery) effects of electrodes implanted around the tibial nerve on the TS motor units properties were reported to cause a small degree of muscle denervation with subsequent reinnervation [52]. Approximately 20% reduction of the number of VGLUT1 terminals contacting LG α-MNs perikarya observed in the sham—stimulated side in our experiment suggests that even relatively short-lasting (4 weeks) enclosure of the tibial nerve in the cuff electrodes might be detrimental. This important observation might partly explain bilateral changes in innervation of α-LG MNs observed in our experiments.

The loss of VGLUT1 synapses on MNs cell bodies and proximal dendrites was reported after transection of peripheral nerve [53, 54]. Moreover, Alvarez and co-authors found that VGLUT1 IF Ia synapses were permanently retracted from lamina IX and did not recover after muscle reinnervation by regenerating sensory and motor axons [53]. However, when operant conditioning was applied after transection of peripheral nerve, it caused compensatory increase in the number of the VGLUT1 IF terminals apposing Sol MNs and led to an increase of the H-reflex [54].

Assuming that lower number of VGLUT1 IF terminals apposing α-LG MNs in the sham-stimulated side, observed in our experiments, indicates some damage of Ia fibers in the tibial nerve, reversal of this effect in the stimulated leg points to successful recovery processes after stimulation of these afferents. This remodeling may be mediated by NT3, that was shown by us to increase after low-threshold stimulation proprioceptive fibers on the stimulated side [10]. NT3 is known to enhance glutamatergic Ia transmission and secure maintenance of Ia terminals abutting on α-MNs [5, 6, 55–57].

Despite the undesirable result of electrode implantation we have found that the effect of one-week low-threshold stimulation undertaken with a three-week delay with respect to the electrode implantation time not only eliminates VGLUT1 input deficit but also compensates significantly a decrease of integrated volume of glutamatergic terminals on soma and proximal dendrites of targeted MNs.

### Stimulation of low-threshold proprioceptive afferents in the tibial nerve increased the number and affected the size and intensity of VGLUT1 IF terminals apposing LG α-MNs

Surprisingly, whereas the number of VGLUT1 terminals abutting on LG MNs increased after stimulation, their mean volume decreased. The shift in the size distribution of VGLUT1 IF terminals might be attributed to increased number of newly formed VGLUT1 IF terminals, which may be smaller and/or functionally immature, containing less vesicular transporter of glutamate, than subpopulations of VGLUT1 of larger sizes [27, 29]. On the other hand, a fraction of small terminals might in part originate from declustered, big terminals. Declustering of VGLUT1 terminals apposing dendritic arbor of α-MNs was reported after injury of the peripheral nerve and subsequent reinnervation by Rotterman and co-authors [23]. Also intraterminal redistribution of VGLUT1-bearing vesicles may have occurred. It was reported that prolonged stimulation of neuromuscular terminals did not affect the number and size of synaptic vesicles but there was a redistribution of vesicles such that they appeared to be channeled in distinct streams to synaptic contacts [58].

If we assume that the intensity of VGLUT1 labeling reflects the number of VGLUT1 molecules in a terminal, an increased VGLUT1 IF intensity in the largest terminals after stimulation may reflect the following processes: (1) a number of synaptic vesicles bearing VGLUT1 is increased, that may result in an increase of neurotransmitter released (quantal content), (2) vesicular volume is increased, as it was shown that expression of VGLUT1 determines vesicular size and glutamate content [27–29]. Therefore, we postulate that increased level of VGLUT1, which determines the efficiency of glutamate loading to synaptic vesicles, reflects functional state of VGLUT1 terminals.

We have to bear in mind that observed changes concern only a small fraction of Ia synaptic terminals on α-MNs, apposing cell bodies and most proximal parts of dendrites. In contrast to VAChT IF synaptic terminals, which are predominantly abutted on the motoneuronal cell bodies, 75%-90% of Ia terminals were found to be located mostly on a proximal half of a dendritic tree [21–24, 40, 41, 59].

While somatic population of Ia VGLUT1 inputs is a minority, these inputs demonstrate high vulnerability to the injury of peripheral nerve, which is higher than that of dendritic Ia inputs, if percentage change in both populations is compared [23]. In rats with peripheral nerve transection, which are stimulated by means of treadmill exercise, Ia terminals reveal ability to reinnervate MN soma, correlating with restoration of H-reflex in exercised rats [60, 61]. The contribution of Ia terminals on dendrites in this restoration is not known, but the studies we quote indicate that somatic subpopulation of Ia terminals contributes significantly to motoneuron functional states [51,57,58].

### Plasticity of cholinergic V0_c_ interneurons induced by electrical stimulation

Increased number of VAChT IF C-terminals abutting upon α-MNs under study after seven days stimulation of low-threshold proprioceptive afferents in the tibial nerve suggests that enhancement of this input to the ankle extensor α-MNs leads to an enrichment of their cholinergic innervation. Similar effect was observed in monkeys (*Macaca nemestrina*) after operant conditioning leading to an increase of the H-reflex in triceps surae (TS) muscle in one leg whereas the other leg was also stimulated but not subjected to the a modification of the H-reflex size [44]. The density of C-terminals contacting TS MNs of the unconditioned but stimulated leg increased by approximately 20% comparing to the control group. Bigger effect (an increase by approximately 80%) appeared on TS MNs of the conditioned leg [44]. These results indicate that both unconditionally and conditionally delivered stimuli addressed to Ia afferents activate spinal interneurons, which have been recently identified as the source cholinergic input to α-MNs [17].

The V0_C_-cholinergic interneurons, located close to the central canal, were identified as the only source of cholinergic innervation of α-MNs by C-terminals [17, 62]. These interneurons exert modulatory, bilateral effects on the α- MNs increasing their excitability [16]. The V0_c_ interneurons receive, among others, indirect input from sensory afferents [17]. Here we confirm that they might be activated by low-threshold proprioceptive Ia input which results not only in the increased number but also in increased intensity of VAChT IF in C-boutons, which we interpret as an increase of VAChT expression reported recently to translate to enhancement of synaptic vesicle neurotransmitter release [30].

Variable latencies of responses of V0c –interneurons to stimulation of the dorsal roots observed by Zagoraiou and co-authors [17] suggest that the sensory input they receive is mediated by spinal interneurons. Among functionally and anatomically identified spinal interneurons, the population of intermediate zone excitatory interneurons receiving input from group I and II muscle afferents should be considered here, as they potentially could mediate the low-threshold proprioceptive input to V0c –interneurons [13]. These interneurons are distinct from Ia inhibitory interneurons, which do not receive input from group II muscle afferents [13, 14]. They are contacted predominantly by terminals expressing VGLUT1, which originate both from the proprioceptive afferents and corticospinal tract [25], and much less frequently by terminals expressing VGLUT2, attributed predominantly to propriospinal input [13]. Although it has not been yet reported that these interneurons are the source of excitation of V0_c_ interneurons, this possibility cannot be excluded (E. Jankowska, *personal communication*). Our study, which shows that the density of cholinergic C-boutons (larger than 5 μm^3^) on LG α-MN cell bodies is comparable to that of VGLUT1 terminals (larger than 1 μm^3^), contributes to investigations indicating an importance of modulation of excitatory input to MNs cell bodies and of the cholinergic partner in MN coverage.

Taken together, our findings provide morphological evidence of synaptic changes on LG α-MNs induced by seven days stimulation of low-threshold proprioceptive afferent fibers in the tibial nerve. The increased number of glutamatergic and cholinergic synaptic terminals abutting on LG α-MNs together with increased aggregate volume of terminals and content of vesicular transporters in some of them indicate that this type of stimulation induces enrichment of excitatory input from both sources to selected group of MNs innervating the ankle extensor muscles. Importantly, this group of MNs was found to be particularly vulnerable to the damage of the spinal cord showing profound decrease in the number of cholinergic boutons apposing extensor but not flexor MNs [18]. Therefore, the proposed pattern of stimulation might be a useful therapeutic tool used to activate selected group of MNs suffering from decreased excitability [54, 63].

Moreover, the same pattern and time of stimulation of low-threshold proprioceptive fibers in the tibial nerve induced clear increase of NT3 in the spinal cord and Sol muscle [10] indicating that observed synaptic rearrangement of both types of inputs might be reinforced and maintained by increased level of this neurotrophin.
